# Author Correction: EGFR core fucosylation, induced by hepatitis C virus, promotes TRIM40-mediated-RIG-I ubiquitination and suppresses interferon-I antiviral defenses

**DOI:** 10.1038/s41467-025-63499-8

**Published:** 2025-08-27

**Authors:** Qiu Pan, Yan Xie, Ying Zhang, Xinqi Guo, Jing Wang, Min Liu, Xiao-Lian Zhang

**Affiliations:** 1 State Key Laboratory of Virology and Hubei Province Key Laboratory of Allergy and Immunology, and Department of Immunology, Wuhan University TaiKang Medical School (School of Basic Medical Sciences), Wuhan, 430071 China; 2 Department of Allergy, Zhongnan Hospital of Wuhan University, Frontier Science Center for Immunology and Metabolism, Medical Research Institute, Wuhan University, Wuhan, 430071 China

**Keywords:** Virus-host interactions, Infection, Hepatitis C virus

Correction to: *Nature Communications* 10.1038/s41467-024-44960-6, published online 22 January 2024

In Figure 7b of this article, a drawing error in the glycosylation pattern of EGFR occurred.

This has now been corrected in both the PDF and HTML versions of the Article.

