# Supplementary material for: EGFR core fucosylation, induced by hepatitis C virus, promotes TRIM40-mediated-RIG-I ubiquitination and suppresses interferon-I antiviral defenses
Source: Nat Commun. 2024 Jan 22;15:652. doi: 10.1038/s41467-024-44960-6 (PMC10803816; doi:10.1038/s41467-024-44960-6)

EGFR core fucosylation, induced by hepatitis C virus, promotes TRIM40-mediated-RIG-I ubiquitination and suppresses interferon-I antiviral defenses

SUPPLEMENTARY INFORMATION

This PDF file includes:

- Supplemental Figures S1-S8
- Supplemental Tables S1-S5
- Uncropped Scans of Blots in Supplementary Figures

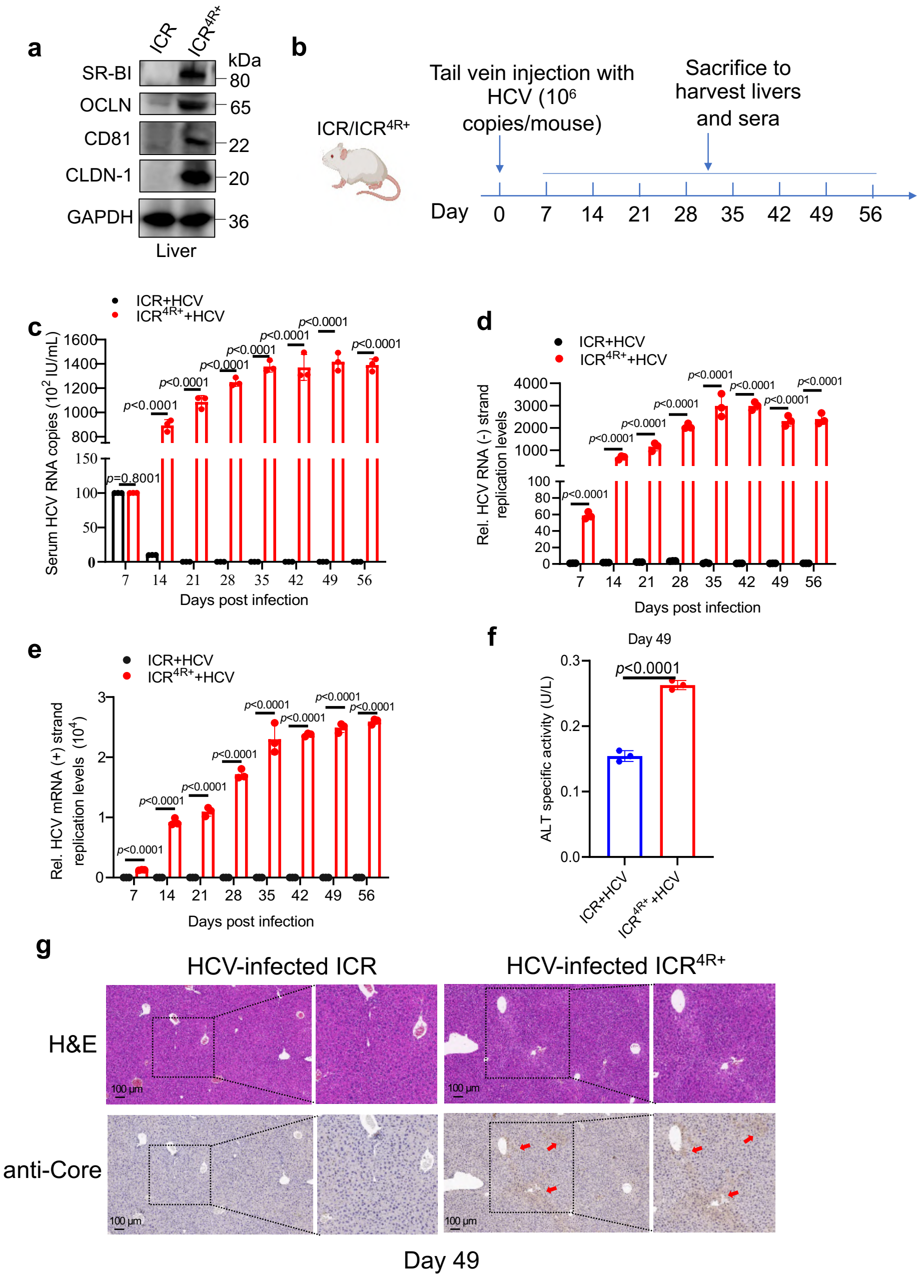

Supplementary Fig. 1. ICR^{4R+} transgenic mouse model for HCV infection. (a) Immunoblot analysis of human SR-BI, OCLN, CD81, and CLDN-1 in the liver tissues of ICR^{4R+}/ICR background mice. (b) Scheme of ICR^{4R+} transgenic mouse model for HCV infection (n = 3 for each time point in each group, 48 mice in total). The figure is created with Biorender.com. (c) The blood samples were collected at different time points during the course of HCV infection. Mouse serum HCV RNA absolute copies were determined using RT-qPCR with standard curve method using TaqMan probe (n = 3 mice per group per study). (d-e) Analysis of relative HCV-negative (-) strand RNA levels using strand-

specific Tth-based RT-qPCR (d) (n = 3 mice per group per study), and relative HCV-positive (+) strand RNA levels by RT-qPCR analysis (e) (n = 3 mice per group per study) in the liver tissues of ICR/ICR^{4R+} mice infected with HCV. (f) Serum ALT levels were measured in ICR/ICR^{4R+} mice at Day 49 post infection. (n = 3 mice per group per study) (g) H&E staining (upper panel) and immunohistochemical staining using anti-Core (lower panel, brown color indicates positively stained region) in liver tissues of HCV-infected ICR/ICR^{4R+} mice. Data are normalized based on *Gapdh* for c-e. Data in all quantitative panels are presented as mean ± SD. Data are representative of two independent experiments. Two-tailed unpaired student's t test was used to assess the statistical difference in c-f.

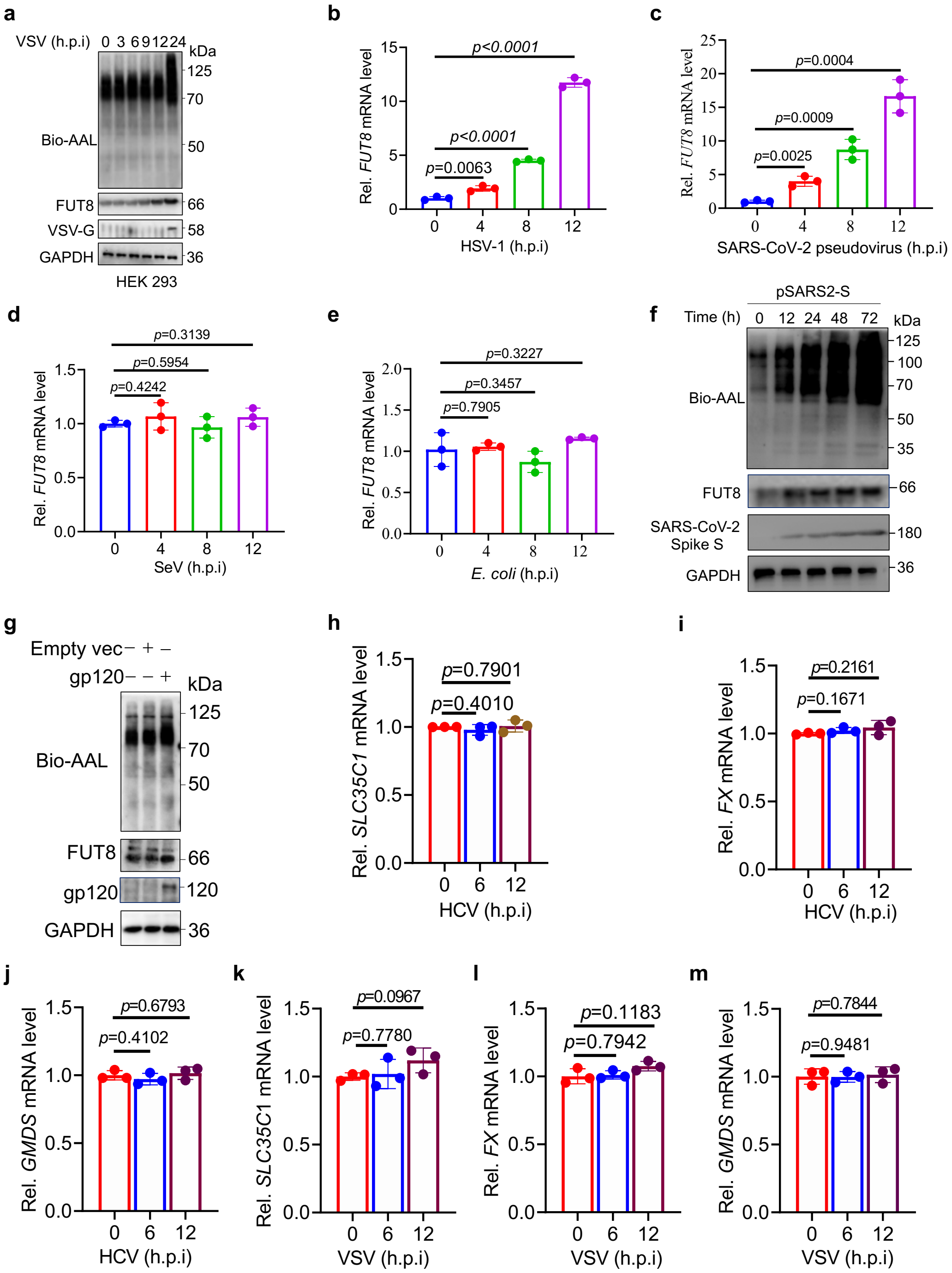

Supplementary Fig. 2. Related to Fig. 1. (a) Lectin blot and immunoblot analysis of core fucosylation, FUT8 and VSV-G expression in lysates of HEK293 cells infected with VSV for the indicated time. (b-e) RT-qPCR analysis of *FUT8* mRNA expression in HEK293T cells with HSV-1 (n = 3 per group per study) (b), SARS-CoV-2 pseudovirus (MOI = 2) (n = 3 per group per study) (c), SeV (MOI = 0.1) (n = 3 per group per study) (d), or *E. coli* (bacterium:cell = 100:1) (n = 3 per group per study) (e) for the indicated time. (f) Lectin blot

and immunoblot analysis of core fucosylation, FUT8 and SARS-CoV-2-Spike expression in lysates of HEK293T cells transiently transfected with pSARS2-S plasmids for the indicated time. (g) Lectin blot and immunoblot analysis of core fucosylation, FUT8 and HIV-gp120 expression in lysates of HEK293T cells transiently transfected with gp120 plasmids for 72 h. **(**h-m**)** RT-qPCR analysis of *SLC35C1, FX, GMDS* mRNA expression in HCV-infected Huh7 cells (MOI = 0.1) (n = 3 per group per study) (h-j) or VSV-infected HEK293T cells (MOI = 0.1) (n = 3 per group per study) (k-m). Data are normalized based on *GAPDH* for b-e and h-m. Data in all quantitative panels are presented as mean ± SD. Data are representative of three independent experiments. Two-tailed unpaired student's t test was used to assess the statistical difference in b-e and h-m.

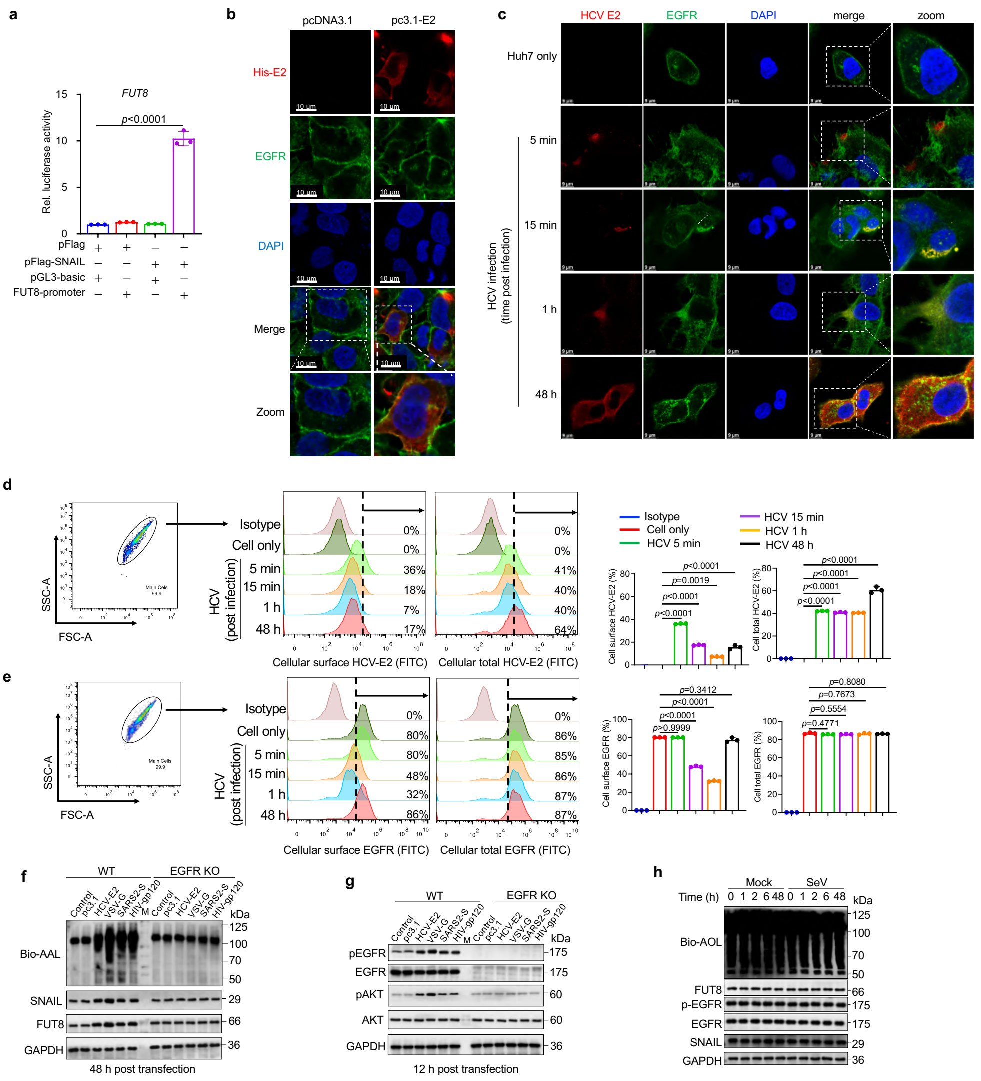

Supplementary Fig. 3. Related to Fig. 2. (a) *FUT8* promoter activity in Huh-7 cells transiently transfected with Flag-vector or Flag-SNAIL plasmids, detected using the luciferase reporter assay. Luciferase activity was analyzed as fold induction (n = 3 per group per study). (b) Huh7 cells were transfected with HCV E2 plasmid or empty vector for 48 h. Cells were fixed and probed for EGFR and His-E2. DAPI was used to stain cellular nuclei. Representative confocal microscopy images are shown. (c) Huh7 cells were infected with HCV (MOI = 10) for the indicated time. Cells were fixed and probed for EGFR and HCV-E2. DAPI was used to stain cellular nuclei. Representative confocal microscopy images are shown. (d-e) FCM analysis im ages of E2 (d) or EGFR (e) in Huh7 cells infected with HCV for indicated time (MOI = 10). Representative FCM image of surface and cellular total E2 (d, left panel) or EGFR (e, left panel) stain. Statistical chart of the percentage of E2 (d, right panel) or EGFR (e, right panel) expression were plotted. Isotype control antibodies were used to define background and non-specific binding signal (n = 3 per group per study). (f-g) Lectin blot for core fucosylation (f), and immunoblot analysis of SNAIL (f), FUT8 (f), p-EGFR (g), EGFR (g), p-AKT (g) and AKT (g) in WT or EGFR KO Huh7 cells transfected with plasmids encoding the indicated viral envelope proteins. M (in f and g) indicates the molecular weight marker. (h) Lectin blot analysis of core fucosylation and immunoblot analysis of FUT8, p-EGFR, EGFR and SNAIL in SeV-infected HEK293 cells (MOI = 0.1) for the indicated time. Data in all quantitative panels are presented as mean ± SD. Data are representative of three independent experiments. Control in f and g: cells transfected with Lipofectamine 2000. Two-tailed unpaired student's t test was used to assess the statistical difference in a (*vs.* pGL3-basic), d (right panel, *vs.* cell only), and e (right panel, *vs.* cell only).

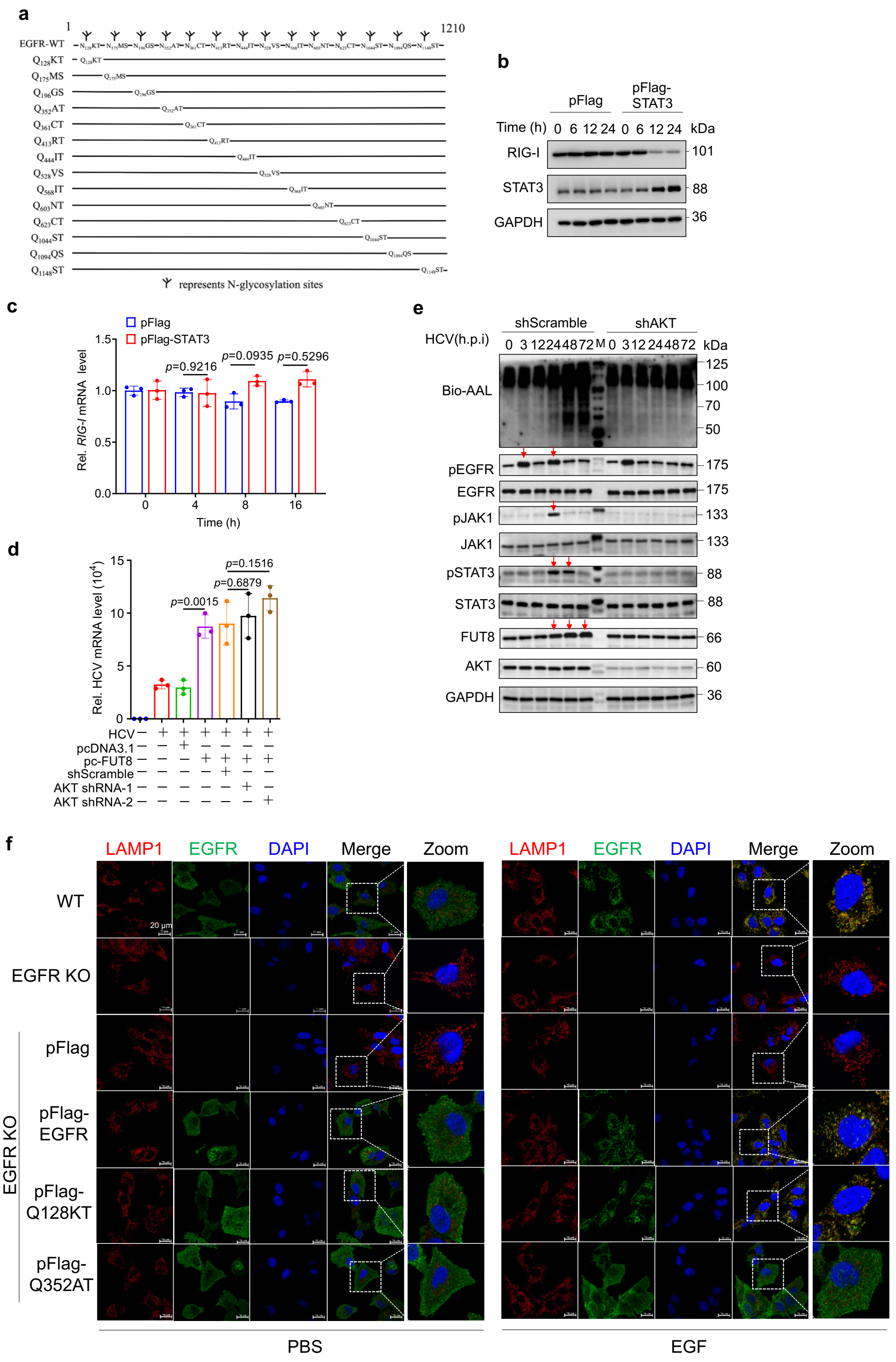

Supplementary Fig. 4. Related to Fig. 3 and 4. (a) Schematic structure of 14 N-glycosylation mutation sites of EGFR. (b) Immunoblot analysis of RIG-I/STAT3 in lysates of Huh7 cells transiently transfected with Flag-vector or Flag-STAT3 plasmids for the indicated time. (c) RT-qPCR analysis of *RIG-I* mRNA expression in of Huh7 cells transiently

transfected with Flag-vector or Flag-STAT3 plasmids for the indicated time (n = 3 per group per study). (d) RT-qPCR analysis of HCV mRNA expression in Huh7 cells transiently transfected with His-vector or His-FUT8 plasmids or co-transfected with AKT shRNA for 48 h, and then infected with HCV (MOI = 0.05) for 48 h (n = 3 per group per study). (e) Huh7 cells were transfected with shRNA-AKT (shAKT) or shScramble for 48 h, and then infected with HCV for 0-72 h (MOI = 0.1). Lectin blot for core fucosylation and immunoblot analysis of p-EGFR, p-JAK1, p-STAT3, FUT8, and AKT in Huh7 cells. (f) Huh7 EGFR KO cells were transfected with WT or Q128KT/Q352AT mutant EGFR plasmid for 48 h followed by incubation 1h on ice with 10 nM (EGF) or PBS, washed and incubated at 37 ℃ for 15 min. Cells were fixed and labelled for EGFR and the lysosomal marker Lamp1. DAPI was used to stain nuclei. Representative confocal microscopy images are shown. Data in all quantitative panels are presented as mean ± SD. Data are representative of three independent experiments. Data are normalized based on *GAPDH* for c and d. Two-tailed unpaired student's t test was used to assess the statistical difference in c-d.

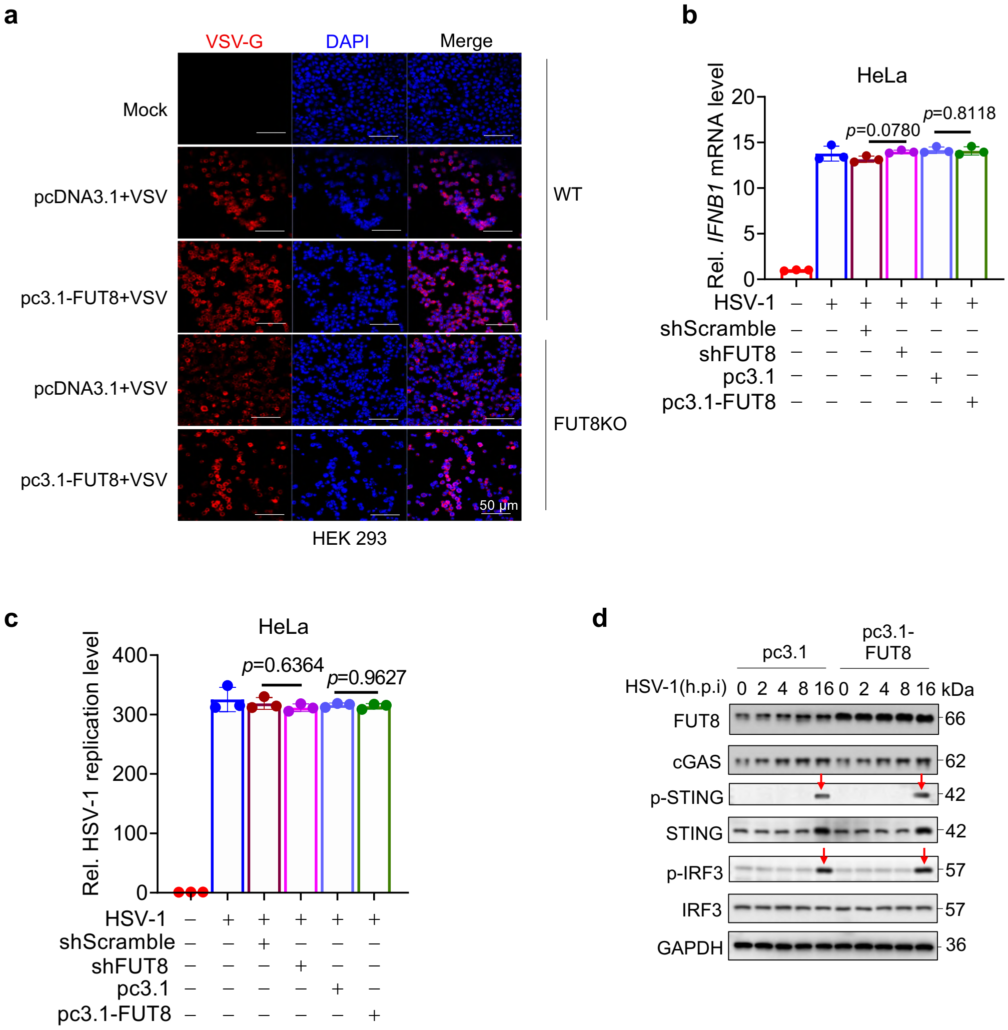

Supplementary Fig. 5. Related to Fig. 5. (a) Confocal microscopy analysis of the effects of FUT8 on VSV-G expression. WT or FUT8KO HEK293 cells were transfected with His-FUT8 for 24 h and then infected with VSV (MOI = 0.01) for 12 h, and labeled with antibodies to the appropriate molecules (scale bar, 50 μm). (b and c) HeLa cells were transfected with the indicated plasmids for 48 h, and then infected with HSV-1 (MOI = 0.01) for 12 h. RT-qPCR analysis of *IFNB1* mRNA expression (b) and HSV-1 replication levels (c) (n = 3 per group per study). Data are normalized based on *GAPDH* for b and c. (d) HeLa cells were transfected with empty vector or pc3.1-FUT8 for 48 h, and then infected with HSV-1 (MOI = 0.1) for the indicated time. Immunoblot analysis of FUT8, cGAS, STING, p-STING, IRF3 and p-IRF3 in HeLa cells. Data in all quantitative panels are presented as mean ± SD. Data are representative of three independent experiments. Two-tailed unpaired student's t test was used to assess the statistical difference in b-c.

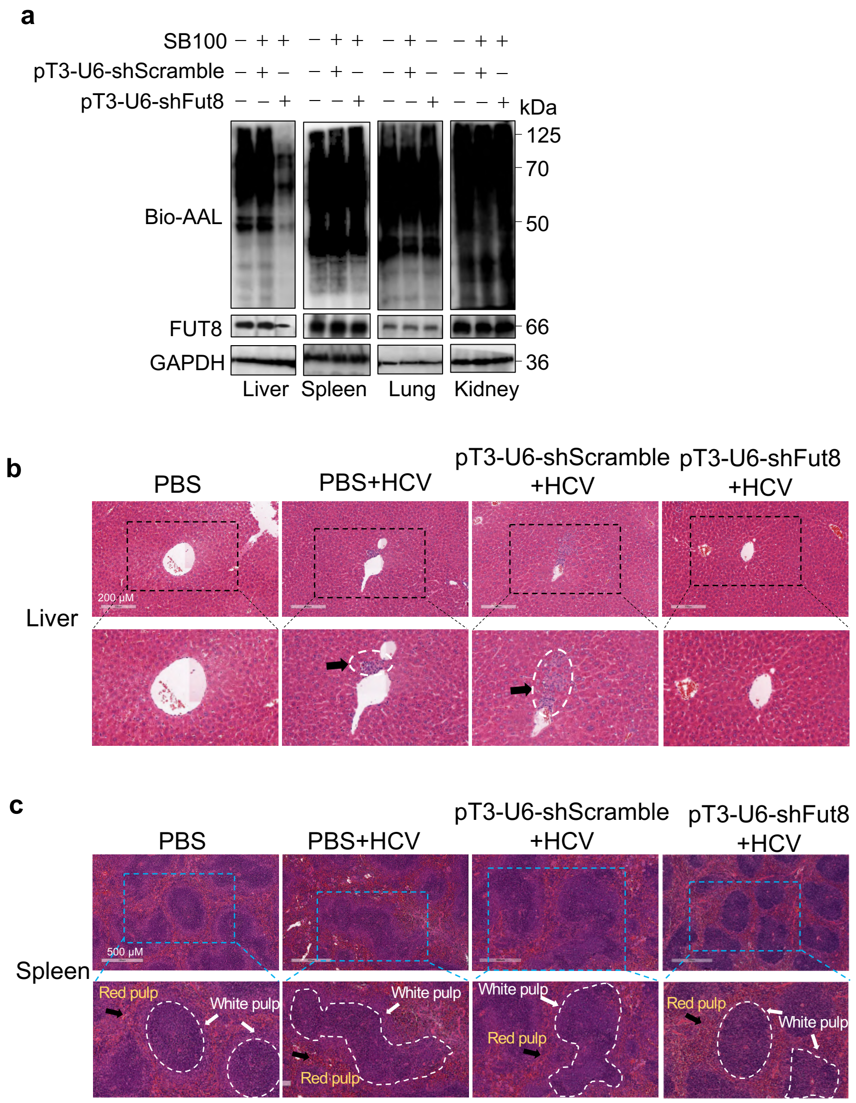

Supplementary Fig. 6. Related to Fig. 6. (a) Lectin analysis of core fucosylation and immunoblot analysis of FUT8 in lysates of liver, spleen, lung and kidney tissues of ICR^{4R+} mice subjected to hydrodynamic injection with the indicated plasmids for 7 d. (b-c) H&E sections of livers (b) and spleens (c) from ICR^{4R+} mice infected with HCV for 30 d. Data are representative of two independent experiments.

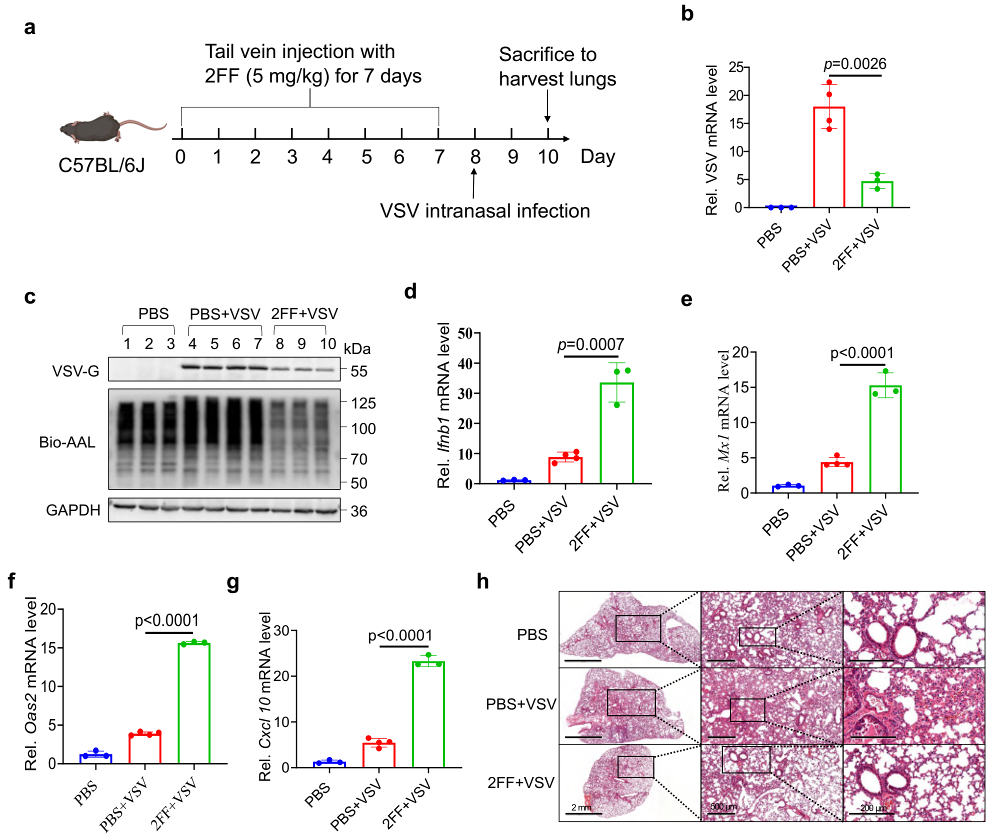

Supplementary Fig. 7. FUT8 inhibitor 2FF suppresses VSV viral RNA replication and viral protein translation in C57BL/6J mouse infection models. (a) Scheme of mouse VSV infection models. The figure is created with Biorender.com. (b, d-g) RT-qPCR analysis of VSV (b), *Ifnb1* (d), *Mx1* (e), *Oas2* (f), and *Cxcl10* (g) in the lungs of C57BL/6J mice treated with 2FF (5 mg/kg) consecutively for 7 d and then infected VSV (1 × 10^6 PFU, per mouse) for 48 h (n = 3 mice for PBS/ 2FF+VSV per group; n = 4 mice for PBS+VSV per group). (c) Lectin blot analysis of core fucosylation and immunoblot analysis of VSV-G protein expression in the lung tissues of C57BL/6J mice treated with 2FF (5 mg/kg) consecutively for 7 d, followed by VSV infection for 48 h. (h) Representative H&E analysis of lungs sections from C57BL/6J mice. Data in all quantitative panels are presented as mean ± SD. Data are representative of two independent experiments. Data are normalized based on *Gapdh* for b, d-g. Two-tailed unpaired student's t test was used to assess the statistical difference in b, d-g.

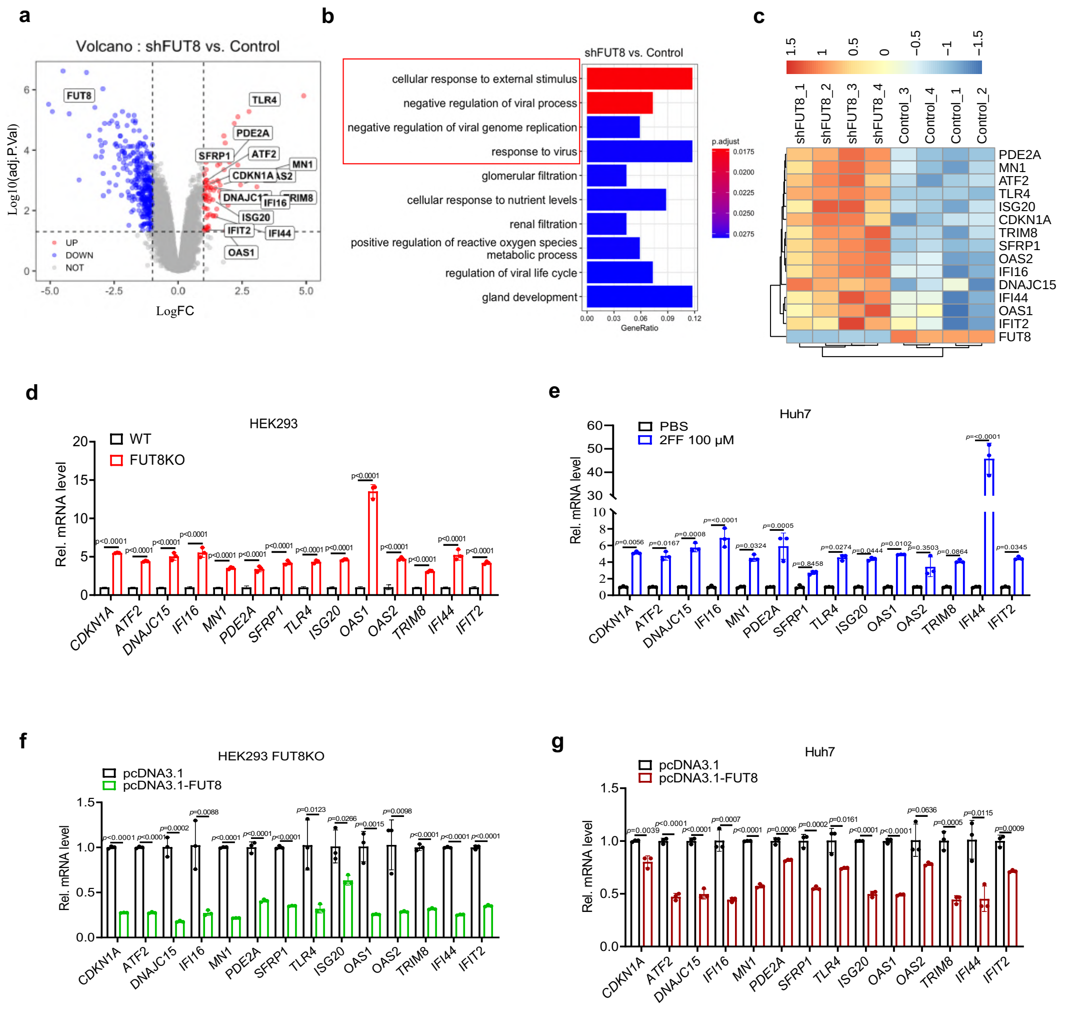

Supplementary Fig. 8. FUT8 negatively regulates anti-viral innate immune response genes. (a) Volcano plot showing differential gene expression for microarray results from FUT8 knockdown versus control human lung cancer cell line CL1-5 cells (GSE42405). Red dots represent 73 upregulated genes (log2(FC) > 1 and adjusted p-value < 0.05) and blue dots represent 342 downregulated genes (log2(FC) < -1 and adjusted *p*-value < 0.05) in FUT8 knockdown versus control CL1-5 cells. Highlighted genes are involved in innate and adaptive immune response pathways. FC: fold-change. (b) A bar chart showing top ten terms in Gene Ontology Biological Processes (GO-BP) term enrichment analysis of upregulated genes in FUT8 knockdown versus control CL1-5 cells. n = 4 biologically independent samples per group. (c) Heatmap showing top 14 DEGs in Supplementary Fig.8a. (d-g) RT-qPCR analysis of the indicated genes from WT and *FUT8* KO HEK293 cells (d), WT and 2FF-treated Huh7 cells (e), *FUT8* rescue in FUT8KO HEK293 cells (f) or

FUT8 overexpression Huh7 cells (g) (n = 3 per group per study). Data in all quantitative panels are presented as mean ± SD. Data in d-g are representative of three independent experiments. Data are normalized based on *GAPDH* for d-g. Two-tailed unpaired student's t test was used to assess the statistical difference in d-g.

Supplementary Tables

Supplementary Table 1. Primers used for plasmids construction in this paper

Name	Forward sequence 5'→3'	Reverse sequence 5'→3'
pcDNA3.1-core	ATGAGCACAAATCCTAAACC	AGCGGAGACCGGGGTGG
pcDNA3.1- p7	ATGGCACTAGAGAAGCTGGTCAT	AGCATAAGCCTGTTGGGG
pcDNA3.1-E1	ATGGCCGAAGTGAAGAACATCAG	CGCGTCCACCCCGGCGG
pcDNA3.1-E2	ATGCGCACCCATACTGTTGGGGG	TGCTTCGGCCTGGCCCA
pcDNA3.1-NS2	ATGTATGACGCATCTGTGCATGG	GAGAAGACTCCACCCCTTGG
pcDNA3.1-NS3	ATGGCTCCCATCACTGCTTATGC	GGTCATGACCTCAAGGTCAG
pcDNA3.1-NS4a	ATGAGCACGTGGGTCCTAGC	GCATTCCTCCATCTCATCAAAAG
pcDNA3.1-NS4b	ATGGCCTCTAGGGCGGCTCT	GCATGGGATGGGGCAGTC
pcDNA3.1-NS5a	ATGTCCGGATCCTGGCTCCG	GCAGCACACGGTGGTATC
pcDNA3.1-NS5b	ATGTCCATGTCATACTCCTGGAC	CCGAGCGGGGAGTAGGA
pcDNA3.1-FUT8	ATGCGGCCATGGACTGGTTCCT	TTTCTCAGCCTCAGGATATGTGGGG
pcDNA3.1-VSV-G	ATGAAGTGCC TTTTGTACTT	TTACTTTCCAAGTCGGTTCA
pFlag-SNAIL	CCGCGCTCTTTCCTCGTCA	TCAGCGGGGACATCCTGAGC
pFlag-STAT3	ATGGCCCAATGGAATCAGCTACAGC	CATGGGGGAGGTAGCG
pGL3-FUT8	ATAAGGAAGGACGGGAGTGA	TCCCTACCTCCTTCCAACTG
pFlag-EGFR-WT	ATGCGACCCTCCGGGACGGCC	TCATGCTCCAATAAATTCACTGCTTTGTGGC
pFlag-EGFR-Q_{128}KT	CTATGATGCACAGAAAACCGGACT	AGTCCGGTTTTCTGTGCATCATAG
pFlag-EGFR-Q_{175}MS	TTCTCAGCCAGATGTCGATGGAC	GTCCATCGACATCTGGCTGAGAA
pFlag-EGFR-Q_{196}GS	CTGTCCCCAAGGGAGCTG	CAGCTCCCTTGGGGACAG
pFlag-EGFR-Q_{352}AT	GACTCACTCTCCATACAAGCTACGAAT	ATTCGTAGCTTGTATGGAGAGTGAGTC
pFlag-EGFR-Q_{361}CT	ACACTTCAAACAATGCACCTCCATC	GATGGAGGTGCATTGTTTGAAGTGT
pFlag-EGFR-Q_{413}RT	GGCCTGAACAAAGGACGGAC	GTCCGTCCTTTGTTCAGGCC
pFlag-EGFR-Q_{444}IT	CGTCAGCCTGCAGATAACATC	GATGTTATCTGCAGGCTGACG
pFlag-EGFR-Q_{528}VS	TCTTGCCGGCAAGTCAGCC	GGCTGACTTGCCGGCAAGA
pFlag-EGFR-Q_{568}IT	AGGCCATGCAGATCACCTG	CAGGTGATCTGCATGGCCT
pFlag-EGFR-Q_{603}NT	TGGGAGAACAGAACACCCTGG	CCAGGGTGTTCTGTTCTCCCA
pFlag-EGFR-Q_{623}CT	TGCCATCCACAGTGCACCTAC	GTAGGTGCACTGTGGATGGCA
pFlag-EGFR-Q_{1044}ST	CAACCAGCCAGCAGTCCACCGT	ACGGTGGACTGCTGGCTGGTTG
pFlag-EGFR-Q_{1094}QS	CTGAATACATACAACAGTCCGTTCCCA	TGGGAACGGACTGTTGTATGTATTCAG
pFlag-EGFR-Q_{1148}ST	CCTGTGTCCAGAGCACATTCG	CGAATGTGCTCTGGACACAGG

Supplementary Table 2. Primers for construction of shRNA/siRNA

Name	Sense 5'→3'	Antisense 5'→3'
pLKO.1-FUT8-1	GCGTTGGATTATGCTCATTCT	AGAATGAGCATAATCCAACGC
pLKO.1-FUT8-2	GCTGGTGTGTAACATCAATAA	TTATTGATGTTACACACCAGC
pLKO.1-EGFR-1	GCAGAGGAATTATGATCTTTC	GAAAGATCATAATTCCTCTGC
pLKO.1-EGFR-2	GCAGATCATCAGAGGAAATAT	ATATTTCCTCTGATGATCTGC
pLKO.1-AKT-1	GCTATTGTGAAGGAGGGTTGG	CCAACCCTCCTTCACAATAGC
pLKO.1-AKT-2	GCTACTTCCTCCTCAAGAATG	CATTCTTGAGGAGGAAGTAGC
pLKO.1-SNAIL-1	GCAACAAGGAATACCTCAGCC	GGCTGAGGTATTCCTTGTTGC
pLKO.1-SNAIL-2	GCAGGACTCTAATCCAGAGTT	AACTCTGGATTAGAGTCCTGC
pLKO.1-JAK1-1	GGTGGAAGTGATCTTCTATCT	AGATAGAAGATCACTTCCACC
pLKO.1-JAK1-2	GCTCTGGTATGCTCCAAATCG	CGATTTGGAGCATACCAGAGC
pLKO.1-STAT3-1	GCAACAGATTGCCTGCATTGG	CCAATGCAGGCAATCTGTTGC
pLKO.1-STAT3-2	GCGTCCAGTTCACTACTAAAG	CTTTAGTAGTGAACTGGACGC
pT3-U6-shFut8	GTTGGATTATGCTCATTCT	AGAATGAGCATAATCCAAC
siTrim40	ACGACUCAAUCGCCGGAGCAGG	CCUGCUCCGGCGAUUGAGUCGU

Supplementary Table 3. Primers for RT-qPCR

Name	Forward sequence 5'→3'	Reverse sequence 5'→3'
FUT1	AGCAACGGCATGGAGTGGTGTA	AAGCCGAAGGTGCCAATGGTCA
FUT2	CTACCACCTGAACGACTGGATG	AGGGTGAACTCCTGGAGGATCT
FUT3	CCGACTACATCACCGAGAAGCT	GAACCTCTCGTAGTTGCTTCTGC
FUT4	GGGTTTGGATGAACTTCGAGTCG	GGTAGCCATAAGGCACAAAGACG
FUT5	ACCTGAGCTACTTTCACTGGCG	TCAGGTGAACCAAGCCGCTATG
FUT6	CCGACTACATCACCGAGAAGCT	GAACCTCTCGTAGTTGCTTCTGC
FUT7	GAATGAGAGCCGATACCAACGC	TAGCGGTCACAGATGGCACAGA
FUT8	GACAGAACTGGTTCAGCGGAGA	GCAGTAGACCACATGATGGAGC
FUT9	TGGAATCAGCCAGCTCTGTGCT	CGTTGTGAGATGGCATCCTTGG
FUT10	CTAACCAGCGACTTCTGACAGC	CCCATCTTTTGGGTGGTAAGCC
FUT11	ACACCTGGCTTTGGCAATGTGG	GTGGATCATGGCAGTGAGAGCT
poFUT1	CCTCCTTGGATTGAGTACCAGC	ATGACCCGATGGTAAGCCTGGA
poFUT2	CCGAGAACCTACTTCACGACCA	TCCGTGGAGTTGAGATGTCTGC
GAPDH	GAAGGTGAAGGTCGGAGTC	GAAGATGGTGATGGGATTTC
Fut1	CTCAATGGTCGCCAAGCCTTCA	GGACTAAGTGCTGCCAAGGTGT
Fut2	AGGCGGTTCAAATGTCCTCACC	GCATATTCGCCCATCTGGTTCC
Fut3	GGCGAATATGCCACGCTGTTTG	CCTTTTGGCTGTGTCGCTATGC
Fut4	GATTGCAGCCTGCGCTTCAACA	AGTCGTGGAGTTCCTTCACCAG
Fut7	CACACTCACCATCCTTATCTGGC	TGGTGGAAGACCACAGCATCAG
Fut8	CCTCAGTCAAACAGATGGAGCAG	ACACCAGCTTCCTGGCTTTGCT
Fut9	CAACCTGACTCTAACTTATCGCC	CAGCACACCAACTTCTCCTTGC
Fut10	CCATCAACAGGACCTTCCAGCA	GGGACTCTTCATGGAAAAGCGC
Fut11	CAGTCCCATTGCGATGTGCCTT	CTGTGTCCTGTAATCGCACGGT
Gapdh	CATCACTGCCACCCAGAAGACTG	ATGCCAGTGAGCTTCCCGTTCAG
HCV	AATCACTCCCCTGTGAGGAAC	TGGTGCACGGTCTACGAGACCTC
VSV	TGCAAGGAAAGCATTGAACAA	GAGGAGTCACCTGGACAATCACT
SeV	CCGAAGTTATGCAAGGCCGAG	GGAGTTCAGGTGACATTCTGG
HSV-1	CATCACCGACCCGGAGAGGGAC	GGGCCAGGCGCTTGTTGGTGTA
IFNB1	TTGTTGAGAACCTCCTGGCT	TGACTATGGTCCAGGCACAG
Ifnb1	GCCTTTGCCATCCAAGAGATGC	ACACTGTCTGCTGGTGGAGTTC
RIG-I	CACCTCAGTTGCTGATGAAGGC	GTCAGAAGGAAGCACTTGCTACC
Mx1	TGGACATTGCTACCACAGAGGC	TTGCCTTCAGCACCTCTGTCCA
Oas2	CACCAAAGTCCTGAAGACCGTC	AGAGTCGTAACTCTCCAGCGAG
Cxcl10	ATCATCCCTGCGAGCCTATCCT	GACCTTTTTTGGCTAAACGCTTTC
CDKN1A	AGGTGGACCTGGAGACTCTCAG	TCCTCTTGGAGAAGATCAGCCG
ATF2	GGTAGCGGATTGGTTAGGACTC	TGCTCTTCTCCGACGACCACTT
DNAJC15	ATGAGTAGGCGAGAAGCTGGTC	GGGTGATTCAAAATCATGACTCTC
IFI16	GATGCCTCCATCAACACCAAGC	CTGTTGCGTTCAGCACCATCAC
MN1	AACCCCAGAACCCCAACAGCAA	TGGACAGACAGGCACTGCAAGT
PDE2A	GCTGGTGAACAAGATCAATGGGC	GCTGCGATACTGAGCCTCATTC
SFRP1	CAATGCCACCGAAGCCTCCAAG	CAAACTCGCTGGCACAGAGATG
TLR4	CCCTGAGGCATTTAGGCAGCTA	AGGTAGAGAGGTGGCTTAGGCT
ISG20	ACACGTCCACTGACAGGCTGTT	ATCTTCCACCGAGCTGTGTCCA
OAS1	AGGAAAGGTGCTTCCGAGGTAG	GGACTGAGGAAGACAACCAGGT
OAS2	GCTTCCGACAATCAACAGCCAAG	CTTGACGATTTTGTGCCGCTCG
TRIM8	GACGTGGAGATCCGAAGGAATG	CAGCCGAACTTCCTCCTTCAGT
IFI44	GTGAGGTCTGTTTTCCAAGGGC	CGGCAGGTATTTGCCATCTTTCC
IFIT2	GGAGCAGATTCTGAGGCTTTGC	GGATGAGGCTTCCAGACTCCAA
SLC35C1	TGCTCACCTGCGGTATCATCA	TCTGGCTTGGTGTGGACCAG
FX	GGTAGCAGATGGAGCTGGACTT	GAAGGTCCAACCCACACACGT
GMDS	ATTGTACGGCGGTCCAGTTCA	GAAGTTGCACTATGGCGATCTCACT

Supplementary Table 4. Probes for EMSA

Probe name	Sense 5'→3'	Antisense 5'→3'
WT	TACACTTCCTGACGTGGCAGGTGAGAGAGTGTGGGT	ACCCACACTCTCTCACCTGCCACGTCAGGAAGTGTA
Mut	TACACTTCCTGACGTGAGAGGGGTCAGAGTGTGGGT	ACCCACACTCTGACCCCTCTCACGTCAGGAAGTGTA

Supplementary Table 5. GeneID in BP_GO_enrichment

Pathway ID	Description	GeneRatio	p.adjust	Gene Symbol
GO:0071496	cellular response to external stimulus	8/68	0.017	CDKN1A/ATF2/DNAJC15/IFI16/MN1/PDE2A/SFRP1/TLR4
GO:0048525	negative regulation of viral process	5/68	0.017	IFI16/ISG20/OAS1/OAS2/TRIM8
GO:0045071	negative regulation of viral genome replication	4/68	0.027	IFI16/ISG20/OAS1/OAS2
GO:0009615	response to virus	8/68	0.022	IFI44/ATF2/IFI16/IFIT2/ISG20/OAS1/OAS2/TRIM8

Uncropped Scans of Blots in Supplementary Figures

Supplementary Fig. 1a

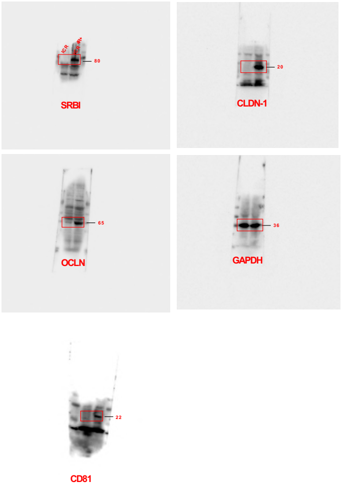

Supplementary Fig. 2g

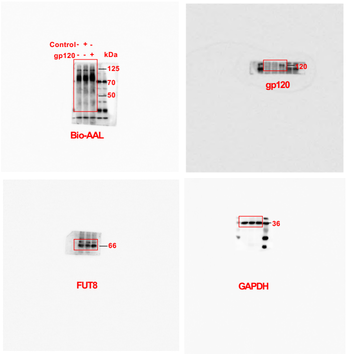

Supplementary Fig. 2a

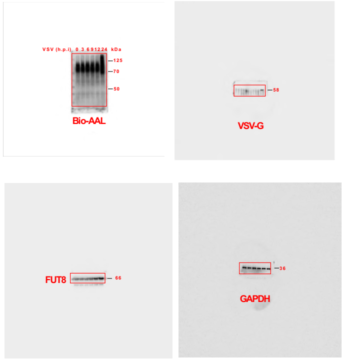

Supplementary Fig. 3c

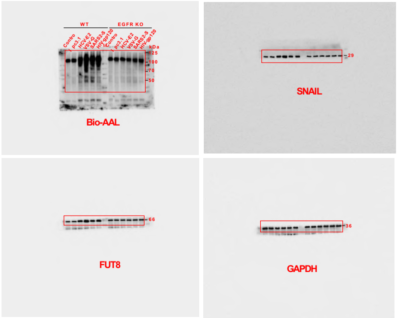

Supplementary Fig3d

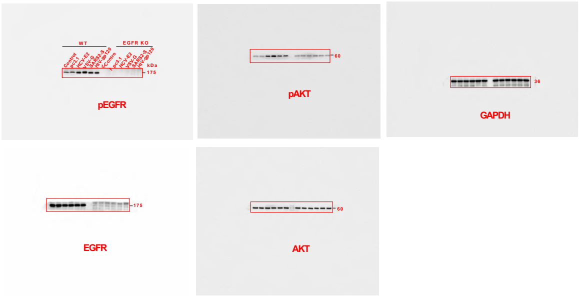

Supplementary Fig. 2f

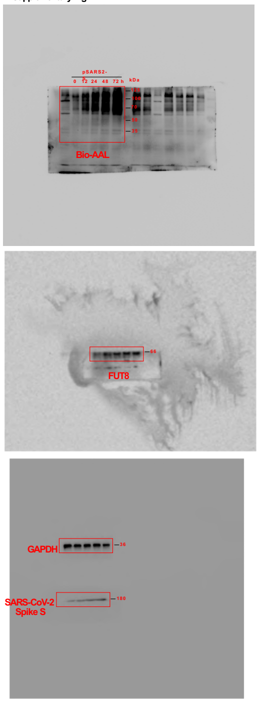

Supplementary Fig. 3e

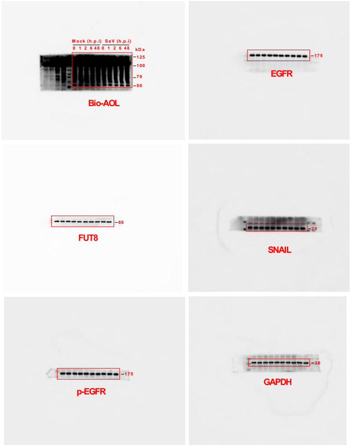

Supplementary Fig. 4b

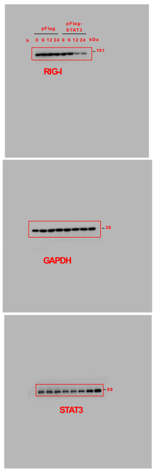

Supplementary Fig. 5d

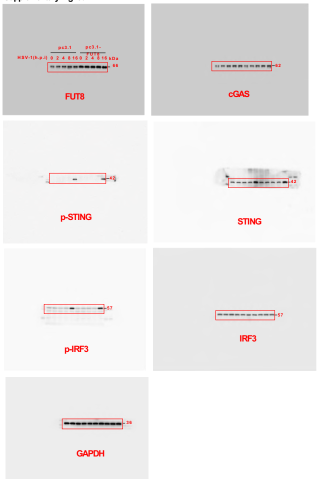

Supplementary Fig. 7c

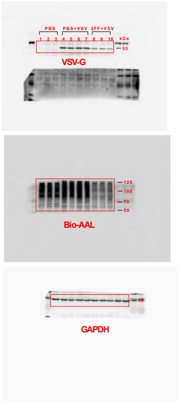

Supplementary Fig. 4e

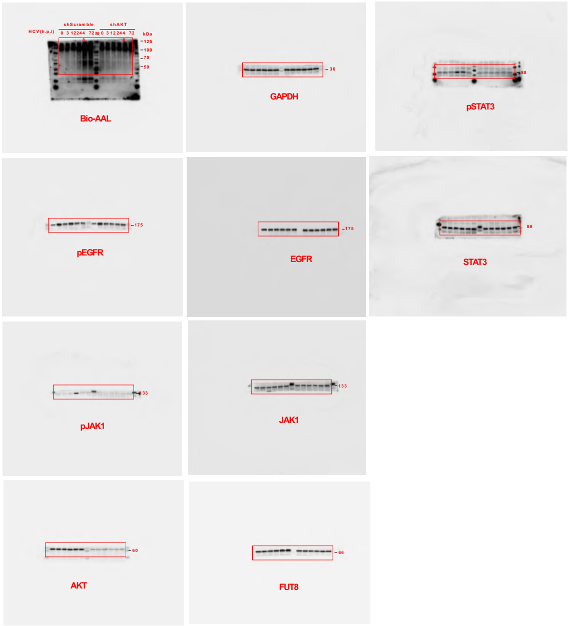

Supplementary Fig. 6a

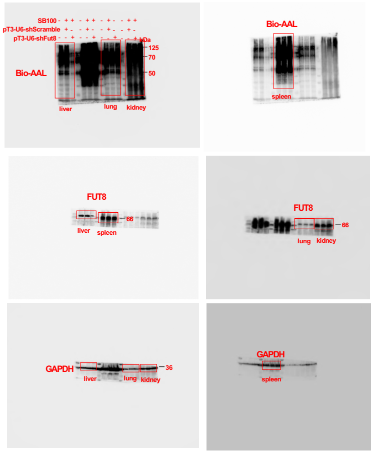

Supplement: Supplementary file 1 — Supplementary Information [file 41467_2024_44960_MOESM1_ESM.pdf]
